# Urinary Biomarkers IGFBP7 and TIMP-2 for the Diagnostic Assessment of Transient and Persistent Acute Kidney Injury in Critically Ill Patients

**DOI:** 10.1371/journal.pone.0169674

**Published:** 2017-01-13

**Authors:** Delphine Daubin, Jean Paul Cristol, Anne Marie Dupuy, Nils Kuster, Noémie Besnard, Laura Platon, Aurèle Buzançais, Vincent Brunot, Fanny Garnier, Olivier Jonquet, Kada Klouche

**Affiliations:** 1 Department of Intensive Care Medicine, Lapeyronie University Hospital, Montpellier, France; 2 Department of Biochemistry, Lapeyronie University Hospital, Montpellier, France; 3 PhyMedExp, Centre National de la Recherche Scientifique (CNRS 9214) - Institut National de la Santé et de la Recherche Médicale (INSERM U-1046), Montpellier University, Montpellier, France; Emory University Department of Medicine, UNITED STATES

## Abstract

**Objective:**

The capability of urinary TIMP-2 (tissue inhibitor of metalloproteinase) and IGFBP7 (insulin-like growth factor binding protein)—NephroCheck Test (NC) = ([TIMP-2] x [IGFBP7]) / 1000)—to predict renal recovery from acute kidney injury (AKI) has been poorly studied. The aim of this study was to assess the performance of measurements of ([TIMP-2] x [IGFBP7]) / 1000) over 24 hours to differentiate transient from persistent AKI.

**Methods:**

Of 460 consecutive adult patients admitted to the ICU, 101 were prospectively studied: 56 men, 62 (52–71) years old. A fresh urine sample was collected at H_0_, H_4_, H_12_ and H_24_ to determine ([TIMP-2] x [IGFBP7]) / 1000) levels. Areas under the curves of Delta NC H_4_-H_o_ and H_12_-H_4_ and serum creatinine (sCr) for detection of AKI recovery were compared.

**Results:**

Forty-one (40.6%) patient were diagnosed with AKI: 27 transient and 14 persistent AKI. At admission (H_0_), AKI patients had a significantly higher NC score than patients without AKI (0.43 [0.07–2.06] vs 0.15 [0.07–0.35], p = 0.027). In AKI groups, transient AKI have a higher NC, at H_0_ and H_4_, than persistent AKI (0.87 [0.09–2.82] vs 0.13 [0.05–0.66] p = 0.035 and 0.13 [0.07–0.61] vs 0.05 [0.02–0.13] p = 0.013). Thereafter, NC level decreased in both AKI groups with a Delta NC score H_4_-H_0_ and H_12_-H_4_ significantly more important in transient AKI. Roc curves showed however that delta NC scores did not discriminate between transient and persistent AKI.

**Conclusion:**

In our population, absolute urinary levels of NC score were higher at early hours after ICU admission (H_0_ and H_4_) in transient AKI as compared to persistent AKI patients. NC variations (Delta NC scores) over the first 12 hours may indicate the AKI’s evolving nature with a more significant decrease in case of transient AKI but were not able to differentiate transient from persistent AKI.

## Background

In critically ill patients, the occurrence of an acute kidney injury (AKI) is frequent and bears a bad prognosis especially when it requires renal replacement therapy (RRT) [1–4]. At early stage, diagnosing and foreseeing the nature of a renal dysfunction—transient or persistent- are a key challenge for routine clinical practice but are deemed difficult. RIFLE, AKIN and KDIGO staging systems have certainly prompted intensivists to follow hourly diuresis and to take into account small creatinine rise for the diagnosis of kidney dysfunction, but enable neither early diagnosis nor transient or persistent AKI diagnosis [3, 5].

Kidney damage is associated with tubular cells lesions and retention of protein waste in the tubular lumen [6]. An abnormal presence of these proteins would be a better marker of kidney lesions and would precede glomerular filtration rate (GFR) decrease and serum creatinine (sCr) increase. Indeed, creatinine rise is rather slow and may occur in other conditions than tubular damage including cardio- and hepato-renal syndromes or obstructive AKI. Several studies validated the clinical value of new biomarkers in AKI detection [7, 8]. Some of them, like plasma cystatin C, are GFR markers, while others, like the urinary cystatin, Neutrophil gelatinase-associated lipocalin [9, 10], urinary interleukin 18 [11] or KIM-1 [12] do mark tubular impairments. These new markers have been successfully tested in numerous clinical conditions including postsurgical cardiology, and admission to the emergency room [10, 13]. The studies conducted in ICUs led however to discordant results due to heterogeneous studied populations and several confounders [7, 14]. Indeed, none of the mentioned biomarkers are routinely used and no recommendation has been made about their use in daily practice to optimize the patient’s care [15].

The use of the combination of two new markers, TIMP-2 (tissue inhibitor of metalloproteinase) and IGFBP7 (insulin-like growth factor binding protein), seems to improve the identification of patients at risk of AKI at 12 hours compared with previous biomarkers [16]. These preliminary results may have however to be confirmed by further investigations. Moreover, these studies did investigate the prediction of AKI occurrence in ICU but without foreseeing its scalability [16]. To the best of our knowledge, very few studies have evaluated ([TIMP-2] x [IGFBP7])/1000) urinary measurements, the AKIRisk/NephroCheck (NC) Kit’s (Astute Medical, San Diego, CA—USA) performance to differentiate transient from persistent AKI. We therefore conducted this study to assess the capability of the urinary measurements of TIMP-2 and IGFBP7, within the first 24 hours in ICU, to early detect AKI and to differentiate transient from persistent AKI.

## Patients and Methods

This observational, prospective, single-center study has been conducted in a medical ICU at the Lapeyronie University Hospital of Montpellier, France and was approved by the Ethic Committee of this institution. A free, informed and signed approval was collected from patients or their relatives by the investigator.

### Population

From March 2014 to May 2015, consecutive adult patients admitted to our ICU were prospectively included in the study. Exclusion criteria included age <18 years, previous chronic renal failure stage >3, stage-c cirrhosis, progressive neoplasia, organ transplant, AKI of obstructive, glomerular or vascular etiology, tumor lysis syndrome, severe rhabdomyolysis, stage-3 AKI according to KDIGO scoring [5], nephrotoxic treatment within the 72 hours before admission, corticotherapy > 1mg/kg, and presumed life expectancy of <48 hours. A bladder catheter was inserted for diuresis monitoring and urine sampling, as well as an arterial catheter for blood pressure monitoring and blood sampling.

### Data collection and study design

Baseline patient characteristics were recorded including age, gender, and comorbidities. Severity of illness was determined using the simplified acute physiology score II (SAPSII), and the Sequential Organ Failure Assessment (SOFA) score. Main therapeutics including type and quantity of fluids, mechanical ventilation, and administration of vasopressor drugs, and RRT were listed.

Blood and urines samples were collected at admission (H_0_), and 4 (H_4_), 12 (H_12_), 24 hours (H_24_) later for blood determination of urea, creatinine (sCr), electrolytes and urine determination of electrolytes.

Fresh urines samples were also taken at each time for AKIRisk/NC score measurements. AKIRisk/NC score results in the measurement of two markers: TIMP-2 and IGFBP7 and is valued according to the formula: NC = ([TIMP-2] X [IGFBP7]/1000). The 2 markers have been measured thanks to an Astute 140 meter^®^ (Astute Medical, San Diego, CA—USA) converting fluorescent signals from each of the two immunoassays. The NC test (Astute Medical, San Diego, CA—USA) requires a fresh urine sample of at least 100 μl. Samples were either immediately processed, the result coming after 20 minutes, or later on with sample storage and freezing at -20°C. In both cases, investigators were blinded to the results of NC scoring. All values for ([TIMP-2] x [IGFBP7])/1000)-NC- are reported in units of [(ng/ml)^2^/1000]. In the validation study, a cut-off of AKIRisk/NC score: ([TIMP-2] x [IGFBP7])/1000)>0.3 [(ng/ml)^2^/1000] was found to be predictive of AKI within 12 hours [16].

### Definitions

AKI was defined as urine volume < 0.5 ml/kg/hour for 6 hours or/and a sCr increase ≥ 26.4 μmol/L or a sCR increase of 1.5 times the baseline value (KDIGO≥1) [5]. Baseline sCr was defined as the usual value of sCr preceding the admission within at least 7days or as the nadir sCr after recovery of renal function. Transient and persistent AKI were defined by the recovery -or not- of renal function at the latest day 5 as previously reported [17] and according to our practice. Recovery of renal function was defined as a return to a sCR value <1.5 times the baseline value or < 26.4 μmol/L above the baseline value with reversal of oliguria and/or return to the baseline sCr. Persistent AKI was defined as a steady or higher AKI KDIGO stage and/or persistence of oliguria after day 5. Glomerular filtration rate was calculated by using the CKD-EPI equation [18].

### Statistical analysis

Statistical analyses were performed using the software R 3.1.0 (R Foundation for Statistical Computing, Vienna, Austria). We first performed a descriptive analysis by computing the frequencies and the percents for categorial data; medians, quartiles (1st quartile-3rd quartile) and extreme values for continuous data. We also checked for the normality of the continuous data distribution using the Shapiro-Wilk's tests. The population was subdivided in two groups according to the occurrence of AKI: no AKI vs AKI. AKI group was then subdivided in two subgroups: transient and persistent AKI. The univariate analysis was performed using two-tailed Student t-test for continuous variables, Fisher and Chi 2 tests for categorial variables or two-tailed Mann-Whitney-Wilcoxon’s test when appropriate. Receiver-operating characteristic curves (ROCs) were plotted to determine the prognostic values of the AKI markers AKIRisk/NC score and creatininemia, and area under the curves (AUC) values were compared using the DeLong’s nonparametric approach [19]. The primary endpoint was AKI recovery within 5 days. A p-value less than 0.05 was considered statistically significant.

## Results

### Study population

**Fig 1:**
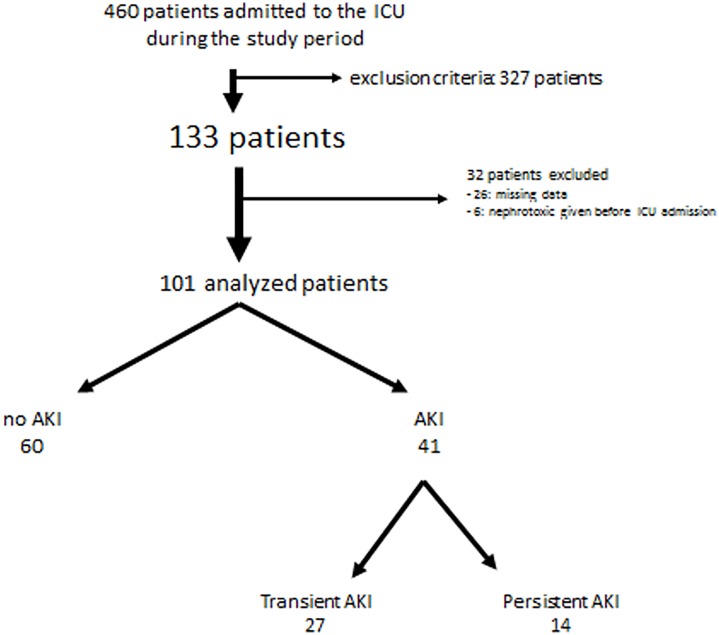
Flow chart of the study population.

During the study period, 460 patients were admitted to the ICU, and 133 of them were included. Among these patients, 32 patients were excluded (26 for missing NC dosages, and 6 for nephrotoxic treatments administered prior to ICU admission) leading to a complete analysis of 101 patients (Fig 1). Table 1 summarizes the main patient characteristics. Median sCr level at admission was 84 μmol/l. More than half of the patients were treated with vasoconstrictive drugs and 2/3 of them were intubated and ventilated. Median duration of ICU stay was 5 days and ICU mortality rate was 17%.

**Table 1:** Baseline characteristics of the studied population and differences between AKI and no AKI groups and between transient and persistent AKI groups of patients.

	*Population*, *n = 101*	*no AKI*, *n = 60*	*AKI*, *n = 41*	p*	*Transient AKI*, *n = 27*	*Persistent AKI*, *n = 14*	p**
**Age, median [IQR], years**	62 [52–71]	59 [47–68]	70 [61–77]	*<0*.*05*	67 [57–72]	73 [70–79]	*<0*.*05*
**Men, n (%)**	56 (55)	29 (48)	27 (66)	*0*.*10*	18 (67)	9 (64)	*0*.*21*
**Comorbidities, n (%)**							
HTA	44 (43.5)	19 (32)	25 (61)	*<0*.*05*	12 (44)	13 (93)	*<0*.*05*
Cardiac failure	30 (29.7)	13(22)	17 (41)	*<0*.*05*	7 (26)	10 (71)	*<0*.*05*
≥ 2 comorbidities	35 (34.6)	15 (25)	20 (49)	*<0*.*05*	9 (33)	11 (78)	*<0*.*05*
**GFR, median [IQR], ml/min/1.73m**^**2**^	85 [68–109]	98 [76–117]	78 [63–90]	*<0*.*05*	82 [70–94]	64 [50–80]	*<0*.*05*
**SAPS II, median [IQR]**	46 [35–57]	43 [30–55]	52 [45–69]	*< 0*.*05*	51 [44–64]	52 [46–71]	*0*.*65*
**SOFA, median [IQR]**	6 [4–9]	5 [4–9]	8 [6–10]	*<0*.*05*	6 [6–9]	8 [7–10]	*0*.*31*
**MV, n (%)**	69 (68.3)	44 (73)	25 (61)	*0*.*21*	14 (52)	11 (79)	*0*.*22*
**Vasopressive drugs, n (%)**	56 (55.4)	24 (40)	32 (78)	*<0*.*05*	21 (78)	11 (79)	*0*.*35*
**RRT, n (%)**	6 (5.9)	0	6 (15)	*<0*.*05*	0	6 (43)	*<0*.*05*
**LOS, median [IQR], days**	5 [3–10]	4.5 [3–9]	6 [3–11]	*0*.*12*	5 [3–9]	10 [6–24]	*<0*.*05*
**Mortality, n (%)**	17	7(11)	10 (24)	*0*.*11*	2 (7)	8 (57)	*<0*.*05*

AKI: acute kidney injury; IQR: interquartile 25–75; GFR: glomerular filtration rate; SAPS-II: simplified acute physiology score II, SOFA: sequential organ failure assessment; MV: mechanical ventilation; RRT: renal replacement therapy, LOS: length of stay.

* differences between no AKI and AKI patients;

**differences between transient and persistent AKI patients

### AKI, transient and persistent AKI occurrence

During ICU stay, AKI occurred in 41 patients (40.6%). Profiles of patients with (n = 41) or without (n = 60) AKI are displayed in Table 1. AKI patients were older (70 vs 59 years), had more comorbidities and a more altered baseline kidney function than others. They were also more severe as reflected by higher SAPSII and SOFA scores and more frequently treated with vasoconstrictive drugs. However, ICU mortality rate was not higher (24% vs 11%, p = 0.11) and ICU length of stay was not extended. Among AKI patients, 27/41 (66%) were detected with a transient AKI and 14/41 (34%) with a persistent AKI. Although older and with more comorbidities, persistent AKI patients were comparable to those with transient AKI in terms of severity. RRT was initiated for 6 patients that developed a persistent AKI. The occurrence of persistent AKI definitively increased ICU mortality (57% vs 7%) and extended ICU length of stay (10 vs 5 days) (Table 1).

### AKIRisk/NC score analysis for the differentiation between transient and persistent AKI

At ICU admission, AKI patients have a higher NC score-([TIMP-2] x [IGFBP7])/1000)—with a median level > 0.3 [(ng/ml)^2^/1000] as compared to no AKI patients. At the 4th hour, NC score was slightly comparable in both groups; it noticeably decreased in AKI group after fluid challenge (Table 2).

**Table 2:** Comparison between no AKI and AKI groups at ICU admission and 4 hours later.

	*No AKI*, *n = 60*	*AKI*, *n = 41*	*p*
**H0**			
Creat median [IQR], μmol/l	65 [53–80]	137 [109–196]	<0.05
Mechanical ventilation, n (%)	41 (68)	17 (41)	<0.05
Vasopressive drugs, n (%)	18 (30)	19 (46)	0.17
AKIRisk/NC score, median [IQR], (ng/ml)^2^/1000	0.15 [0.07–0,35]	0.43 [0.07–2,06]	0.027
**H4**			
Creat. median [IQR], μmol/l	61 [51–78]	129 [98–189]	<0.05
Diuresis, median [IQR], ml/h	30 [20–53]	21 [15–55]	0.31
Mechanical ventilation, n (%)	43 (72)	21 (51)	0.15
Vasopressive drugs, n (%)	25 (42)	22 (54)	0.21
AKIRisk/NC score, median [IQR], (ng/ml)^2^/1000	0.08 [0.04–0.29]	0.09 [0.04–0.78]	0.67

AKI: acute kidney injury; Creat: creatininemia; KDIGO: Kidney Disease Improving Global Outcomes; NC score: NephroCheck score.

IQR: interquartile 25–75; p: differences between AKI and no AKI patients.

**Fig 2:**
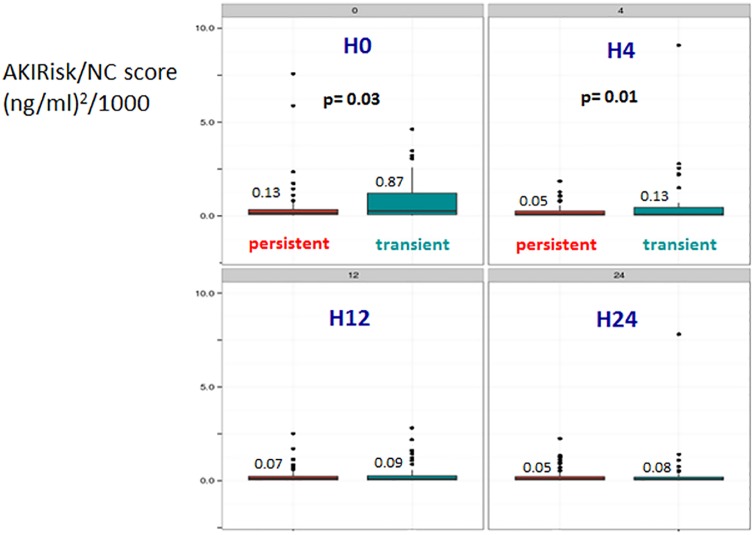
AKIRisk/NephroCheck score at ICU admission (H_0_) and the following 24 hours: comparison between transient and persistent AKI patients. AKI: acute kidney injury; NC: NephroCheck.

Regarding AKI groups (Table 3), NC score was higher in transient AKI than in persistent AKI group at admission (0.87 vs 0.13 [(ng/ml)^2^/1000], p = 0.035). Conversely, sCr level and kidney function’s grading were comparable between groups. At the 4th hour, sCr level, kidney function grading, and urine volume remained comparable between both groups while NC score decreased in both groups but remained higher in transient AKI group (Fig 2). Over the next hours (H_12_ and H_24_), sCr, KDIGO score, urine volume, and NC scores did not enable to differentiate the two groups.

**Table 3:** Comparison of transient and persistent AKI groups at ICU admission and the following 24 hours.

	*Transient AKI*, *n = 27*	*Persistent AKI*, *n = 14*	*p*
**H0**			
Creat. median [IQR], μmol/l	149 [106–205]	120 [109–145]	0.72
KDIGO score, median [IQR]	1 [1–2]	1 [0–1]	0.84
Mechanical ventilation, n (%)	9 (33)	8 (57)	0.35
Vasopressive drugs, n (%)	13 (48)	6 (43)	0.42
AKIRisk/NC score, median [IQR], (ng/ml)^2^/1000	0.87 [0.09–2.82]	0.13 [0.05–0.66]	0.035
**H4**			
Creat. median [IQR], μmol/l	134 [100–194]	122 [99–143]	0.65
Diuresis, median [IQR], ml/h	20 [14–28]	36 [20–64]	0.17
KDIGO score, median [IQR]	1 [1–2]	1 [0–1]	0.54
Mechanical ventilation, n (%)	13 (48)	8 (57)	0.48
Vasopressive drugs, n (%)	15 (56)	7 (50)	0.45
AKIRisk/NC score, median [IQR], (ng/ml)^2^/1000	0.13 [0.07–0.61]	0.05 [0.02–0.13]	0.013
**H12**			
Creat. median [IQR], μmol/l	130 [96–179]	129 [93–161]	0.78
Diuresis, median [IQR], ml/h	30 [22–41]	31 [20–46]	0.76
KDIGO score, median [IQR]	1 [0–2]	1 [0–1]	0.84
Mechanical ventilation, n (%)	14 (52)	8 (57)	0.46
Vasopressive drugs, n (%)	17 (63)	10 (71)	0.39
AKIRisk/NC score, median [IQR], (ng/ml)^2^/1000	0.09 [0.05–0.59]	0,07 [0.02–0.21]	0.32
**H24**			
Creat. median [IQR], μmol/l	114 [81–156]	128 [94–176]	0.26
Diuresis, median [IQR], ml/h	41 [27–47]	28 [16–53]	0.11
KDIGO score, median [IQR]	1 [0–1]	1 [1–2]	0.39
Mechanical ventilation, n (%)	13 (48)	8 (57)	0.86
Vasopressive drugs, n (%)	14 (52)	11 (79)	0.22
SOFA, median [IQR]	6 [1–8]	7 [6–9]	<0.05
AKIRisk/NC score, median [IQR], (ng/ml)^2^/1000	0.08 (0.06–0.34)	0.05 (0.02–0.09)	0.06

AKI: acute kidney injury; creat: creatininemia; KDIGO: Kidney Disease Improving Global Outcomes; SOFA: Sequential Organ Failure Assessment; NC score: NephroCheck score.

IQR: interquartile 25–75; sd: standard deviation, p: differences between transient and persistent AKI patients.

**Fig 3:**
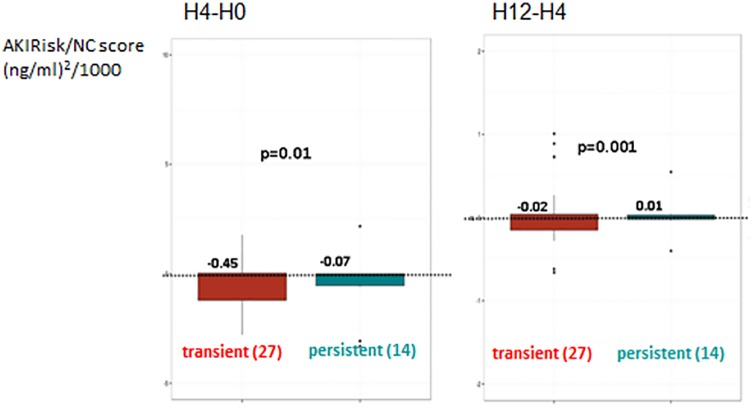
Deltas AKIRisk/NephroCheck score H_4_-H_0_ and H_12_-H_4_: comparison between transient and persistent AKI patients. AKI: acute kidney injury; NC: NephroCheck.

**Fig 4:**
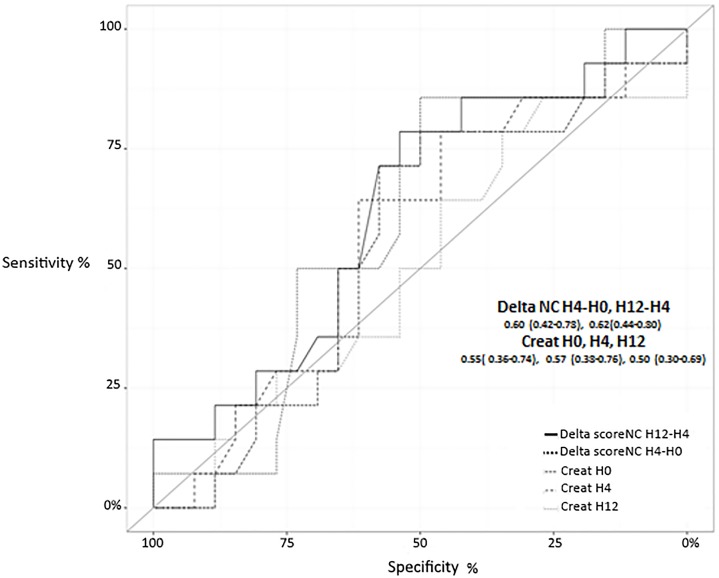
Area under the curves for creatininemia (H_0_, H_4_,H_12_) and for Deltas AKIRisk/NephroCheck score H_4_-H_0_ and H_12_-H_4_ to differentiate between transient or persistent acute kidney injury. AKI: acute kidney injury; NC: NephroCheck.

Analysis of measurements at the successive time point’s showed that NC score quickly decreased after H_0_ to reach a level less than 0.1 in both groups (Table 3, Fig 2). However, Deltas of NC score H_4_-H_0_ (NC score H_4_- NC score H_0_) and H_12_-H_4_ (NC score H_12_- NC score H_4_) were more important in transient AKI group (-0.45 vs -0.07 [(ng/ml)^2^/1000], p = 0.0002 and -0.02 vs 0.01 [(ng/ml)^2^/1000], p<0.0001) as displayed in Fig 3. However, ROC curves displayed on Fig 4 showed that neither H_4_-H_0_ and H_12_-H_4_ Delta NC scores nor sCr discriminate transient from persistent AKI.

## Discussion

In this study, patients with AKI have higher urinary levels of NC score than those without AKI at ICU admission. Also, NC score levels were higher in patients with transient AKI than in those with persistent AKI at ICU admission (H_0_) and 4 hours later (H_4_). The analysis of NC score at different time points showed that NC score decreased in the first 12 hours in case of transient AKI but remained at the same level, or even increased in case of persistent AKI. Deltas NC score H_4_-H_0_ and H_12_-H_4_ were indeed higher in transient AKI but did not enable to differentiate between transient and persistent AKI.

Numerous biomarkers of tubular impairment have been tested and developed to early detect AKI including NGAL, KIM-1, and interleukin 18. These biomarkers have already shown some limitations, especially their lack of specificity. Indeed, they may rise in settings like a non-complicated sepsis without occurred renal function alteration. It may due to their high sensibility to tubular injury and their fast kinetics unlike sCr [7, 14, 20]. Recently, several studies showed the superiority of AKIRisk/NC score- newly developed marker associating two biomarkers: TIMP-2 and IGFBP7- over other markers for an early detection of AKI [16]. A first single-centric study, counting 522 patients in ICU, led to select, among the 340 proteins identified as early markers, the most sensitive and most specific biomarkers of AKI. IGFBP7 and TIMP-2 were already known as having the best performance with an AUC of respectively 0.77 and 0.75. A second multicenter observational study (Sapphire Study) including 728 patients validated the performance of these markers with an AUC for IGFBP7 and for TIMP-2 statistically superior to those of KIM-1 and NGAL. More interestingly, their performance was enhanced when associated. The Sapphire study’s results helped to set up two threshold values of the detection of AKI: AKIRisk/NC score of 0.3 for a better sensitivity (90%) and of 2 for specificity (95%) that were confirmed later [16, 21–23]. In our study, a AKIRisk/NC score at ICU admission was higher for patients with AKI with a median score> 0.3 [(ng/ml)^2^/1000]. Our population was exclusively medical with many comorbidities and AKI is mostly multi-factorial and its origin prior to the admission [3, 4]. Indeed, the type and timing of the tubular aggression are rarely precisely identified conversely to post-operative AKI [24]. Our study shows that the AKIRisk/NC score is associated with a tubular aggression since it is higher in case of AKI but remains <0.2 [(ng/ml)^2^/1000] if no AKI occurs. We observed however a decrease in this score from the fourth hour with medians < 0.1 [(ng/ml)^2^/1000] despite the AKI. This decrease seems discordant especially with studies conducted in post-cardiac surgery [23, 24, 25]. Meersch et al [23] observed a significant increase in the AKIRisk/NC score from the 4th hour, earlier than the rise in creatinine and till the 12th hour after cardiac surgery in the group with AKI. One should however retain that the Bell’s study does not exhibit such a good performance of AKIRisk/NC score in AKI prediction although few patients were included [25].

The main purpose of this study was to evaluate the performance of the NC score to differentiate transient from persistent AKI in the first 24 hours in ICU. Out of the 41 AKI patients, 27 transient AKIs and 14 persistent AKIs were identified. The differentiation between transient AKI and persistent AKI is crucial because it impacts both the care and prognosis [20, 26, 27]. Clinical and biological evidences (FE of sodium, FE of urea, U/P urea and osmolality…) are widely used for that matter but several studies have unveiled their lack of reliability especially in ICU [17, 28–30]. Echo-Doppler measurements of renal vascular resistance seemed to efficiently differentiate the 2 types of kidney impairment but results again disappointed [31, 32]. This emphasizes the value of an early identification and validation of new, performing urinary biomarkers. Previous studies did not enable to select one biomarker in this purpose [7, 9, 14, 33]. In our population, we found that the absolute values of NC score at different times (H_0_ and H_4_) were higher in transient AKI as compared to persistent AKI patients which may seem paradoxical regarding to previous studies. Indeed in a Sapphire’s ancillary study, IGFBP7 was identified as an early prognostic marker of the AKI’s severity, duration and associated mortality [34]. Also, Yamashita showed that TIMP-2 was performing in severe AKI detection and was predictive of poor prognosis [35]. More interestingly, Dewitte et al investigated NC score’s kinetics and recently reported that NC score was significantly higher at H_0_ and H_24_ in persistent AKI patients [36]. The authors concluded to a good predictability of the NC score’s kinetics over the first 24 hours in the recovery of the kidney function after the second day. Here again, the populations are not comparable because the aforementioned study mostly included surgical patients. These discordant results led us to investigate further. We observed a decrease in NC score at H_4_ and the following time measurements, mainly in the group with transient AKI. Moreover, Deltas NC score H_4_-H_0_ and H_12_-H_4_ were higher in transient AKI patients. It was mainly a fluid loading, as if relevant therapeutics, that triggered a NC score decrease in case of transient AKI but has a lesser impact in the case of persistent AKI.

The differentiation between pre-renal or functional AKI and renal or organic AKI was questioned, from 2007 by Bellomo [26, 37], for patients with severe sepsis. The functional and organic AKI may work the same way and only the duration of the degradation would define the impairment’s severity namely its transient or persistent nature [38, 39]. Our results would then comply with this concept. Accordingly, the increase in the NC score at an early stage is a marker of tubular impairment whatever the type of transient or persistent AKI; even higher if AKI is transient. It may suggest that TIMP2 and IGFBP7 are released as an alarm signal of aggression with a greater response in case of hemodynamic changes like functional AKI and that a decrease or stabilization of these biomarkers, mostly dependent on the therapy, would characterize the AKI. Our observations may be put in line with the Zarbock’s observations [40]. In a study of ischemic preconditioning, he demonstrated a rise in ([TIMP-2] x [IGFBP7]) / 1000) levels, but not of NGAL and HMGB-1, for all patients who went through a presurgical ischemic preconditioning versus a control group. Ischemic preconditioning would then induce a significant increase in AKIRisk/NC score in contrast with the other biomarkers, suggesting that the combination of [TIMP-2] and [IGFBP7] is more sensitive to transient hemodynamic variations [41]. The next hours, this rise was corrected for the patients preconditioned and unscathed from AKI but not for the patients who will later suffered from an AKI [40]. However, Deltas NC score H_4_-H_0_ and H_12_-H_4_, as reflected by ROC curve, were not discriminative of transient and persistent AKI in our population.

This study has numerous limitations. It is single-center, prospective but with a quite low number of patients, so it lacked power. This study was conducted exclusively on medical patients and cannot be therefore extrapolated to other surgical wards. Our recruitment was often “second hand”, explaining the severity of patient cases. It made also the results hardly extrapolable to other ICUs. We defined persistent AKI as a non-recovery kidney function at D_5_ while numerous thresholds were used: D_1_ to D_3_ [28, 29, 42, 43]. The D_5_ milestone was also selected because it looks representative of our daily practice. Some authors like Vanmassenhove proceeded the same way [17] while some others like Brown postponed it to D_7_, a timeframe definitely associated with a rise in mortality [30,44].

## Conclusion

The NC score is a marker of tubular lesions with a greater increase in transient AKI as compared to persistent AKI suggesting that TIMP2 and IGFBP7 are more sensitive to hemodynamic changes. The investigation of the NC score’s kinetics in the frame of an AKI may help for a better physiopathological understanding of the AKI and its transient or persistent mechanism. It remains however necessary to confirm these data with larger studies and targeted populations.

## Abbreviations

AKI: acute kidney injury
GFR: glomerular filtration rate
IGFBP7: insulin-like growth factor binding protein
NC: NephroCheck Test
RRT: renal replacement therapy
SAPSII: simplified acute physiology score II
sCr: serum creatinine
SOFA: sequential organ failure assessment score
TIMP-2: tissue inhibitor of metalloproteinase
